# 3D Hierarchically Mesoporous Zinc-Nickel-Cobalt Ternary Oxide (Zn_0.6_Ni_0.8_Co_1.6_O_4_) Nanowires for High-Performance Asymmetric Supercapacitors

**DOI:** 10.3389/fchem.2020.00487

**Published:** 2020-06-15

**Authors:** Muhammad Tayyab Ahsan, Muhammad Usman, Zeeshan Ali, Sofia Javed, Rashad Ali, Muhammad U. Farooq, Muhammad Aftab Akram, Asif Mahmood

**Affiliations:** ^1^School of Chemical & Materials Engineering, National University of Sciences and Technology (NUST), Islamabad, Pakistan; ^2^Department of Materials Science and Engineering, College of Engineering, Peking University, Beijing, China; ^3^School of Materials and Energy, University of Electronic Science and Technology of China, Chengdu, China; ^4^Department of Physics, University of Education, Faisalabad Campus, Faisalabad, Pakistan; ^5^School of Chemical and Biomolecular Engineering, The University of Sydney, Sydney, NSW, Australia

**Keywords:** zinc-nickel-cobalt oxide, hierarchically porous nanostructures, SCs, aqueous asymmetric supercapacitors, high energy density

## Abstract

Increased efforts have been devoted recently to develop high-energy-density supercapacitors (SC) without renouncing their power efficiency. Herein, a hierarchically mesoporous nanostructure of zinc-nickel-cobalt oxide (ZNCO) nanowires (NWs) is constructed by hierarchical aggregation of ZNCO nanoparticles. It is worth noting that cobalt and nickel rich lattice imparts higher charge storage capability by enhanced reversible Faradaic reaction while zinc provides structural stability and higher conductivity. Moreover, particulate nature of ZNCO NWs allows deep diffusion of electrolyte thus enabling reversible charge storage under higher current densities. The as-prepared ZNCO NWs exhibited excellent specific capacitance of 2082.21 F g^−1^ at the current density of 1 A g^−1^ with high stability up to 5,000 charge-discharge cycles. Further, the asymmetric SC device was assembled using ZNCO NWs (ZNCO NWs//MWCNTs) which exhibited high energy density of 37.89 Wh kg^−1^ and excellent capacitance retention up to 88.5% over 1,000 cycles. This work presents ways to construct multi-component high-energy-density materials for next-generation energy storage devices.

**Graphical Abstract:**
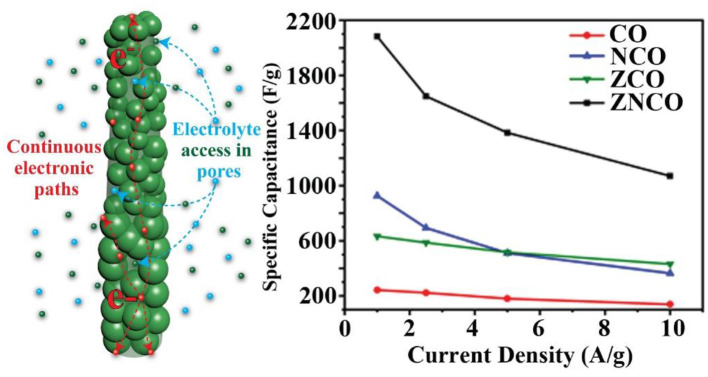
The mixed metal oxides, having a combination of various transition metal-ion species have emerged as promising electrode materials owing to their higher electrochemical activities and relatively high electronic conductivities. Properly tuned nanostructures with hierarchical porosity provides opportunity to tailor high capacity mixed metal oxides.

## Introduction

Supercapacitors (SCs) have attracted enormous interest due to high power density, fast charging/discharging and long service life and have found wide scale applications from portable electronic devices to large road markets (Wang et al., 2012; González et al., 2016; Han et al., 2018). However, the SCs generally exhibit lower energy densities (0-10 Wh Kg^−1^) which limit their applications at commercial scale (Pomerantseva and Gogotsi, 2017; Raza et al., 2018). The issue of relatively lower energy density has been addressed by enhancing either the capacitive performance i.e., increasing specific capacitance or by widening the operating voltage window (Xu et al., 2010; Kim et al., 2015). The specific capacitance and operating window are dependent upon the structure and properties of the constituent materials along with several other factors. Nanotechnology has opened up new horizons with the development of novel materials and Nanostructures. A number of electrode materials have been investigated for enhancing the capacitive performance of the SC devices including carbonaceous nanostructures, metallic species, metal/carbon hybrids etc (Mahmood et al., 2015, 2016; Tabassum et al., 2017, 2018; Jian et al., 2019). Among various electrode materials, the metal based materials have shown promising results owing to reversible Faradaic reactions happening on the metal electrode surface accounting for higher charge storage capability (Xu et al., 2010; Hu et al., 2017; Sun et al., 2018; Zhao et al., 2018; Chen and Lin, 2019; Chen et al., 2019). In particular, the mixed metal oxides, having a combination of various transition metal-ion species have emerged as promising electrode materials owing to their higher electrochemical activities and relatively high electronic conductivities. However, the large-scale applications of mixed metal oxides are limited by poor control over their inherent structure, composition and poor cyclic stability.

To improve the specific capacitance and cyclic stability of mixed metal oxides, the possibility of designing porous chemistries which can allow fast mass diffusion have been proposed recently. However, designing hierarchically porous chemistries to promote electrode/electrolyte interface with excellent control over composition of mixed metal oxides is highly challenging. The existence of meso- and micro- pores are generally considered to be beneficial for fast mass diffusion and improved electrode/electrolyte contact, respectively (Pan et al., 2006; Yuan et al., 2011; Wu et al., 2015; Fang et al., 2018; He et al., 2018; Sahoo et al., 2018). Moreover, the presence of various metallic species in crystal lattice provides a number of active sites for Faradaic redox reaction, enhances conductivity and offers the opportunity to operate under high current densities (Wu et al., 2015). Hence, designing mixed metal oxide nanostructures with excellent control over their structure is critical in order to realize the maximum specific capacitance with high cyclic life. Moreover, a good control over chemical composition is another challenge when tailoring the inherent structure of nanomaterials which needs to be addressed (Xiong et al., 2014; Lin et al., 2016; Maitra et al., 2017; Sahoo and Shim, 2017; Yan et al., 2017; Azman et al., 2018). Recently, trimetallic oxides have been proposed for SC applications owing to their enhanced electrochemical activities and longer stability (Zhao et al., 2015, 2016; Ma et al., 2016; Sun et al., 2018; Wang et al., 2018; Alqahtani et al., 2019). However, developing optimum nanostructure for trimetallic oxides with single phase product having optimum pore structure for enhanced and fast charge storage is highly challenging.

Herein, we report controlled synthesis of hierarchically mesoporous Zinc-Nickel-Cobalt oxide (Zn_0.6_Ni_0.8_Co_1.6_O_4_, ZNCO) nanowires (NWs) by a facile hydrothermal method. Keeping in mind the high specific activity of Co, the Zn and Ni species have been introduced into the Co rich lattice to obtain Zn_0.6_Ni_0.8_Co_1.6_O_4_ with needle-like NW structure formed by hierarchical aggregation of ZNCO nanoparticles. The mesopores arising from hierarchical hetero-aggregation of nanoparticles provided shorter diffusion pathways for electrolytes, thus enabling the reversible operation at higher charge/discharge rates. Owing to unique mesoporous structure, the developed material exhibited excellent capacitive performance in 6 M KOH electrolyte delivering specific capacitance of 2082.21 F g^−1^ at scan rate of 1 A g^−1^ with remarkable cyclic stability of 120% after 5,000 cycles. The asymmetric device assembled using ZNCO NWs as positive and MWCNTs as negative electrode exhibited a high specific capacitance of 121.268 F g^−1^ at 1 A g^−1^, superior energy density of 37.89 Wh kg^−1^ at the corresponding power density of 749.8 W kg^−1^ and excellent cyclic stability of 90% after 1,000 cycles. The exceptional electrochemical performance of ZNCO NWs, as well as its high performance as an asymmetric device with wide operating window, indicates that it is one of the most promising candidates for future energy storage devices.

## Experimentation

### Synthesis

Co(NO_3_)_2_·6H_2_O>99% was purchased from UNICHEM, Zn(NO_3_)_2_·6H_2_O>98%, Ni(NO_3_)_2_·6H_2_O >97% pure, CO(NH_2_)_2_>98% and NH_4_F were purchased from DAEJUNG, KOH was purchased from MERK, nafion@deflourinated resin solution and absolute ethanol 99.8% pure were purchased from Sigma Aldrich and all chemical were utilized without further purification. The ZNCO NWs were synthesized by the simple hydrothermal method. In first step 0.3 M zinc nitrate hexahydrate (Zn(NO_3_)_2_·6H_2_O), 0.3 M nickel nitrate hexahydrate (Ni(NO_3_)_2_·6H_2_O), 0.6 M cobalt nitrate hexahydrate (Co(NO_3_)_2_·6H_2_O), 1.2 M urea (Co(NH_2_)_2_) and 0.4 M ammonium fluoride (NH_4_F) were added to the 50 ml of distilled water with continuous stirring for 2 h. The homogeneous mixture was transferred into the 100 ml of Teflon lined stainless steel autoclave. The autoclave was kept in the oven at 130^O^C for 5 h. Then precipitates were washed several times with distilled water, and absolute ethanol and precipitates were filtered out by vacuum filtration. Afterwards, the sample was dried at 60°C for 6 h and then annealed at 350°C at heating rate of 2^o^C/min in a muffle furnace for 2 h to obtain the crystal structure of ZNCO NWs. For comparison, nickel cobalt oxide (NCO), zinc cobalt oxide (ZCO), nickel oxide (NO), cobalt oxide (CO) were prepared by the same process.

### Characterization

FEI Tecnai F30 (transmission electron microscope, TEM) and field emission scanning electron microscope (MIRA3 TESCAN) were used for the morphological characterization of the product. Moreover, selected area selected area electron diffraction (SAED) and high resolution TEM (HRTEM) analysis was also carried out by FEI Tecnai F30. Elemental composition and oxidation states of the material was analyzed by Energy dispersive spectroscopy detector equipped in (MIRA3 TESCAN) and X-ray photoelectron spectroscopy (XPS), Kratos Axis Ultra with monochromatized Al Kα radiation (1486.6 eV). X-ray Diffraction Analysis (STOE diffractometer) was used to determine the crystal structure of ZNCO NWs. In order to find the surface area of ZNCO NWs surface area analyzer Micromeritics Gemini VII 2390 was utilized.

### Electrochemical Analysis

Electrochemical properties of the synthesized materials were carried out on electrochemical work station. A three electrode setup was used where the sample was first prepared by adding catalyst material &10 μl nafion in ethanol and sonicated for 1 h then drop cast on glassy carbon electrode of 3 mm diameter having surface area of 7.065 × 10^−6^ m^2^ and dried at 60°C, Platinum (Pt) wire as a counter electrode (CE) and Ag/AgCl as a reference electrode (RE). Cyclic voltammetry (CV) was performed at 100, 50, 20, and 10 mV s^−1^ scan rate with operating potential window of −0.1 to 0.5 V. Galvanostatic charge/discharge (GCD) test was done at 1, 2.5, 5, and 10 A g^−1^. Electrochemical impedance spectroscopy was executed from 100 to 1 MHz. All electrochemical tests were performed in 6M KOH solution.

Specific capacitance was calculated by following equation

$$\begin{array}{l} Cs=\;\frac{I\;*\;t}{m\;*\;\Delta V} \end{array}$$

Here, “t” is discharge time, “I” represents current and “m” is mass of active material, Δ*V* is the voltage drop during discharge.

Electrochemical tests of asymmetric SC (ASC) were carried out by using two electrode assembly. Electrochemical cell was constructed using ZNCO NWs as positive electrode and MWCNTs as negative electrode. All the tests were performed at a wide operating window of 0 V to 1.5 V. Asymmetric SC performance was strongly influenced by the charge balance between the positive and negative electrodes. Following charge balance equation utilized to calculate the loading of mass on electrode is

$$\begin{array}{l} \frac{m^{+}\;}{m^{-\;}}=\frac{c^{-}\times {\Delta V}^{-}}{c^{+}\times \;{\Delta V}^{+}} \end{array}$$

Where m is the mass (g) of the electrodes. C is the specific capacitance (F g^−1^), and Δ*V* is the potential window. Optimized mass to charge ratio for this asymmetric cell is 0.5.

## Results and Discussions

**Figure 1:**
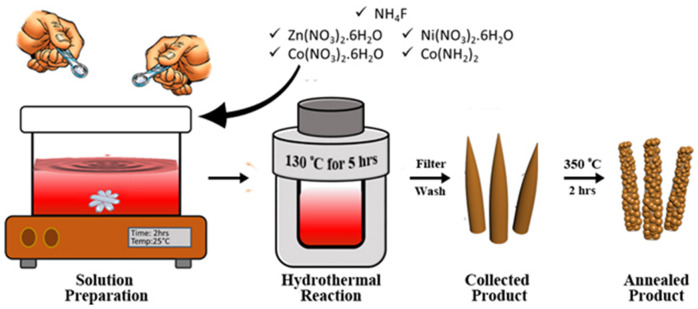
Schematic illustration of the synthesis process of ZNCO. A homogenous solution of precursors is prepared by magnetic stirring at room temperature. Hydrothermal reaction produces needle like structure which are then heat treated obtain hierarchically mesoporous ZNCO NWs.

**Figure 2:**
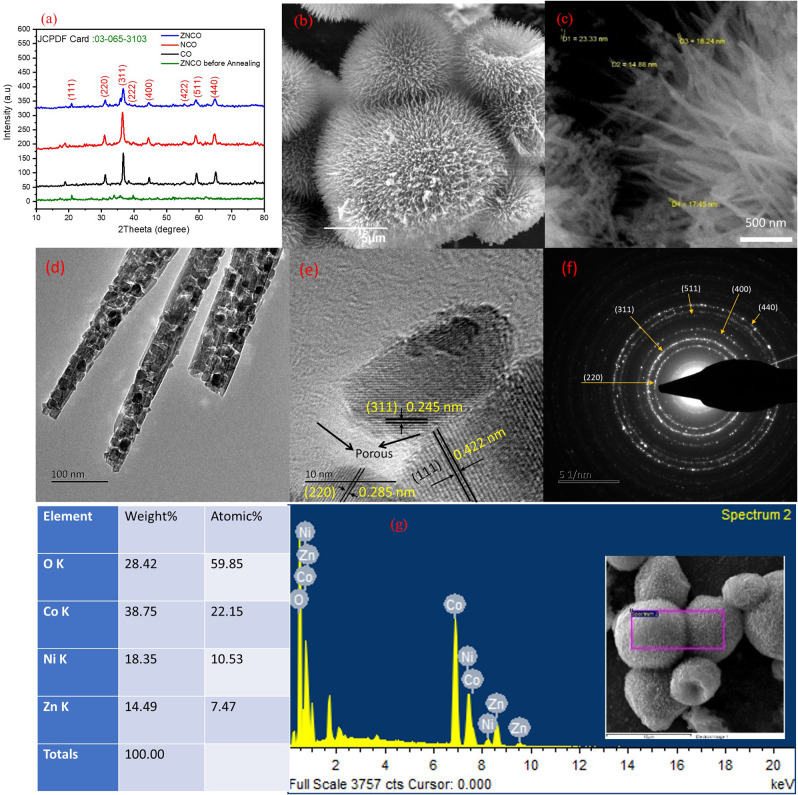
**(a)** XRD of Co, NCO, ZNCO and ZNCO before annealing **(b)** SEM of Hierarchically arranged ZNCO nanostructures **(c)** FESEM image of ZNCO NWs **(d)** TEM Image of ZNCO NWs **(e)** HRTEM of ZNCO crystal **(f)** SAED of ZNCO **(g)** EDS spectrum of ZNCO NWs.

The hierarchically mesoporous ZNCO NWs were synthesized by a simple two-step methodology using hydrothermal treatment followed by calcination in air as depicted in Figure 1. The XRD analysis was performed to determine the crystal structure of ZNCO NWs and the results are presented in Figure 2a and XRD of MWCNTs in Figure S4. The analysis of pure Co_3_O_4_ revealed that diffraction peaks correspond well with standard card (JCPDS: 03-065-3103) and the crystal structure belongs to the cubic and space group of Fd-3m having (111), (220), (222), (400), (422), (511), (440) planes. The analysis of NCO (Ni doped Co_3_O_4_) and Zn/Ni (Zn/Ni codoped Co_3_O_4_) exhibited similar diffraction pattern to pure Co_3_O_4_ confirming successful addition of Ni and Zn into the crystal lattice of Co_3_O_4_ without any phase impurity. All diffraction peaks of doped samples correspond well with standard card (JCPDS: 03-065-3103) confirming the phase purity in the product. It is important to note that the diffraction peaks are slightly shifted toward higher angle in comparison to reference pattern which is due to the difference in the cations of the Zn, Ni and Co (Wu et al., 2018). The crystallite size of ZNCO NWs was calculated by using the Scherrer Formula and the average crystallite size of ZNCO NWs was found to be ~8.1 nm. The Field emission scanning electron microscope (FESEM) was utilized to investigate the morphology of the developed products and the results are presented in Figures 2b,c and Figure S5. The FESEM analysis revealed that the CO, NCO and ZNCO NWs are composed of homogeneous 3D needle-like structures (Figure 2b and Figures S1, S2). High magnification analysis revealed that the NWs consisted of an average diameter of *ca*. 18–20 nm (Figure 2c). The existence of 3D needle-like morphology ensures large surface area and enhanced electrode-electrolyte interface for better charge storage.

To obtain more insights into the internal structure, the TEM analysis was carried out (Figure 2d). It is obvious from TEM results that the ZNCO NWs are formed by aggregation of crystalline particles. It can be assumed that the calcination at 350°C leads to growth of crystalline particles and removal of unreacted species which leaves various open spaces among particles and leads to the creation of porous region. This generation of pores not only ensures electrolyte infiltration leading to enhanced electrode/electrolyte interface but also guarantees fast mass diffusion whereby the developed electrode material could be utilized at higher charge/discharge rates. The high magnification analysis revealed existence of large pores in the NWs as shown in Figure 2e and Figure S10. Furthermore, the HRTEM analysis revealed interplanar d-spacing of 0.245, 0.422, and 0.285 nm corresponding to (311), (111) and (220) planes matched with the observed d-spacing of the XRD pattern (Figure 2e). It is worth mentioning that the crystallite size observed with HRTEM clearly matched with the size calculated from Scherrer Formula. The SAED pattern was recorded to gain insights into the crystal structure of ZNCO NWs and presented in Figure 2f. The SAED pattern affirmed the polycrystalline nature of ZNCO NWs with d-spacing of 0.285,0.249,0.207,0.158, and 0.146 nm referring to (220), (311), (400), (511) and (440) planes. The EDS analysis was used to estimate the elemental composition in ZNCO NWs, and the Zinc, Nickel, Cobalt, and oxygen were found to be in atomic weight percent of 7.47, 10.53, 22.15, 59.85, respectively (Figure 2g and Figure S11). The elemental composition further validates the existence of Zn_0.6_Ni_0.8_ Co_1.6_O_4_ phase in ZNCO NWs.

**Figure 3:**
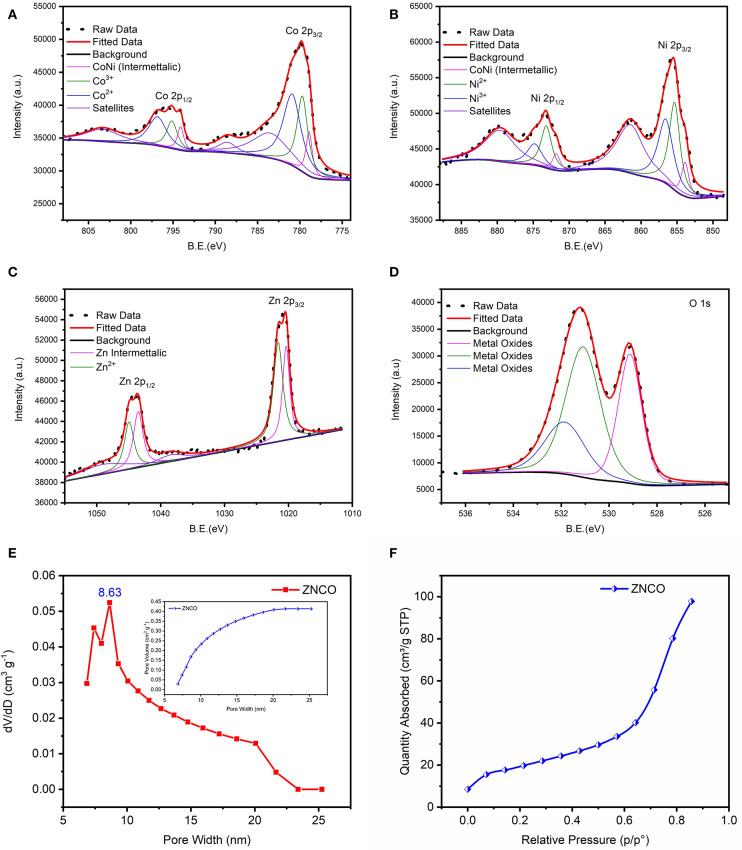
XPS spectra of ZNCO NWs **(A)** Co 2p **(B)** Ni 2p **(C)** Zn 2p **(D)** O 1s **(E)** BET pore volume and pore size distribution of ZNCO NWs **(F)** N_2_ absorption isotherm at 77 K of ZNCO NWs.

The XPS analysis was carried out to examine the detailed composition and oxidation state of ZNCO NWs. Low-resolution XPS survey verifies the presence of Zn, Ni, Co and O elements in a typical ZNCO NWs sample as shown in Figure S9. The Co 2p spectrum (Figure 3A) represents two broad peaks of Co 2p_1/2_ at high binding energy of 795.18 eV and Co 2p_3/2_ at low binding energy of 779.83 eV. The deconvolution of Co indicates the existence of Co^2+^ (in tetrahedral sites) and Co^3+^ (in octahedral sites) as well two shakeup satellites at 783.74 eV and 788 eV (Figure 3A) (Wang et al., 2018). Moreover, the spin-orbit splitting of 15.35 eV was observed for Co 2p_1/2_ and Co 2p_3/2_ indicating the coexistence of both Co^2+^/Co^3+^ (Chen et al., 2015). The deconvolution of Ni 2p spectrum indicates the presence of both Ni^2+^ and Ni^3+^ (Figure 3B; Wu et al., 2015; Sahoo and Shim, 2017). Similarly, the deconvolution of Zn exhibited two peaks of Zn 2p_3/2_ at 1020.5 eV and Zn 2p_1/2_ at 1043.5 eV in as shown in Figure 3C (Zhao et al., 2016). The deconvolution of O1s exhibited three oxygen metal bond peaks at binding energy of 529.1 eV, 531.1 eV and 531.9 eV correspond to zinc oxide, cobalt oxide, and nickel oxide, respectively, in Figure 3D (Bao et al., 2014; Ding et al., 2014; Bhagwan et al., 2019). All these results show that ZNCO NWs contain Co^3+^, Co^2+^, Ni^3+^, Ni^2+^, and Zn^2+^. The inherent porosity of the electrode materials plays key role in determining their respective charge storage capacity. The porosity of the developed products was investigated using Brunauer–Emmett–Teller (BET) analysis and presented in Figure 3E and Figure S7. Average pore diameter of ZNCO is 8.63 nm centered in Figures 3E,F. The ZNCO NWs exhibited pore volume of (0.447 cm3 g^−1^) and much higher surface area of 68.3066 m^2^ g^−1^ in comparison to CO (33.7236 m^2^ g^−1^) and ZCO (47.8949 m^2^ g^−1^). However, the ZNCO NWs surface area was slightly lower than NCO (75.0092 m^2^ g^−1^). The large specific area of ZNCO NWs results in the electrolyte transfer through the electrode material efficiently and increase the reactive sites which result in superior electrochemical performance.

**Figure 4:**
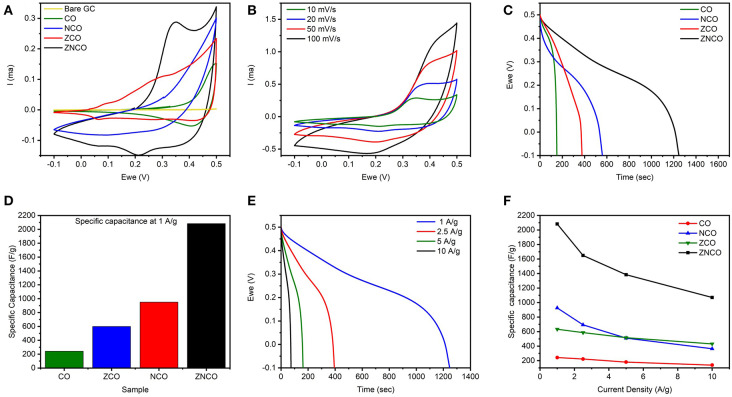
**(A)** CV Comparison of CO, NCO, ZCO, ZNCO and Bare glassy carbon at 10 mV s^−1^ **(B)** CV curves of ZNCO nanostructures at different scan rates **(C)** Discharge curves comparison of CO, NCO, ZCO, ZNCO at 1 A g^−1^ **(D)** Specific Capacitance comparison of CO, ZCO, NCO and ZNCO at 1 A g^−1^ **(E)** GCD cycles of ZNCO nanostructures at different current densities **(F)** Specific capacitance of CO, ZCO, NCO and ZNCO at different current densities.

The designed ternary metal oxide NWs were studied as electrode for SC because of unique structural and compositional features. The ZNCO NWs are expected to exhibit more stability in comparison to CO and other counterparts because of the presence of structure stabilizing species in the form of Zn and Ni which not only stabilize the crystal lattice but also provide higher electronic conductivity. Highly porous nature of NWs provides large electrode/electrolyte interface while mesoporosity ensure presence of large channels for fast diffusion of electrolytes, thus enabling the operation under higher charge/discharge rate. Moreover, the small size of ZNCO nanoparticles (~15–20 nm) in the NW allows deep diffusion of electrolyte into the crystal lattice and ensures utilization of all active species for charge storage. The electrochemical performance was studied in 3-electrode system using ZNCO NWs as working electrode, Pt wire as counter and Ag/AgCl as reference electrode in 6 M KOH electrolyte solution. Figure 4A shows the CV analysis of various products in the voltage window of −0.1–0.5 V at the scan rate of 10 mV s^−1^. Among various products, the ZNCO NWs exhibited the highest area under CV curve and a sharp redox peak with strong oxidation (0.35 V) and reduction (0.22 V) as shown in Figure 4A. While the other products in comparison CO NCO and ZCO show less area under the curve as well as only oxidation or reduction peak. Both the oxidation and reduction phenomena in the ZNCO sample during CV testing leads toward the better electrochemical performance as compared to CO NCO ZCO samples. The electrochemical behavior of ZNCO NWs was further analyzed by CV analysis at different scan rates (10, 20, 50, and 100 mV s^−1^) and the results are presented in Figure 4B. Increasing the scan rate from 10 to 100 mV s^−1^ caused slight peak shift to higher potential and the peaks are not clearly visible as the limitation of diffusion reaction with the increase of scan rate and less redox reaction occurs as less diffusion occurs so the redox peaks become less prominent at higher scan rates.

To investigate actual capacitance values, the Galvanostatic charge-discharge (GCD) analysis was carried out to gain more insights into the performance of the electrode materials. As shown in Figure 4C, all products exhibit a clear plateau in the charge/discharge profile clearly indicating the undergoing redox reaction at current density of 1 A g^−1^. However, ZNCO NWs exhibits longest discharge time indicating high charge storage capacity in comparison to CO, ZCO and NCO which is consistent with CV analysis. Figure 4D represents the comparison of capacitive performance of ZNCO NWs with CO, ZCO and NCO. It is quite clear that ZNCO NWs exhibits much larger capacitance of 2082.21 F g^−1^ at a current density of 1 A g^−1^ as compared to the capacitive performances arising from CO (242.88 F g^−1^), ZCO (633.06 F g^−1^) and NCO (925.7 F g^−1^) as summarized in Table S2. The higher performance for ZNCO NWs can be attributed to relatively larger number of active sites for Faradaic redox reaction, larger surface area and particulate morphology which allows deep diffusion of electrolyte thus enabling the active sites deep inside the particle to participate in the charge storage process. The rate capability of the ZNCO NWs was further studied at various current densities and the results are presented in Figures 4E,F and Table S3. Rate capability of CO,NCO and ZCO are presented in Figures S3d–f. It is interesting to note that the charge-discharge curves retain non-linear nature even at high current densities of 10 A g^−1^. The presence of well-defined plateau at higher current densities clearly indicate the reversible Faradaic redox reaction even at much higher discharge rates. Owing to highly reversible Faradaic redox reaction at higher current densities, the ZNCO NWs exhibited excellent capacitance retention of 1648.37, 1363.86, 1070 F g^−1^ at 2.5, 5, and 10 A g^−1^ as shown in Figure 4F.

**Figure 5:**
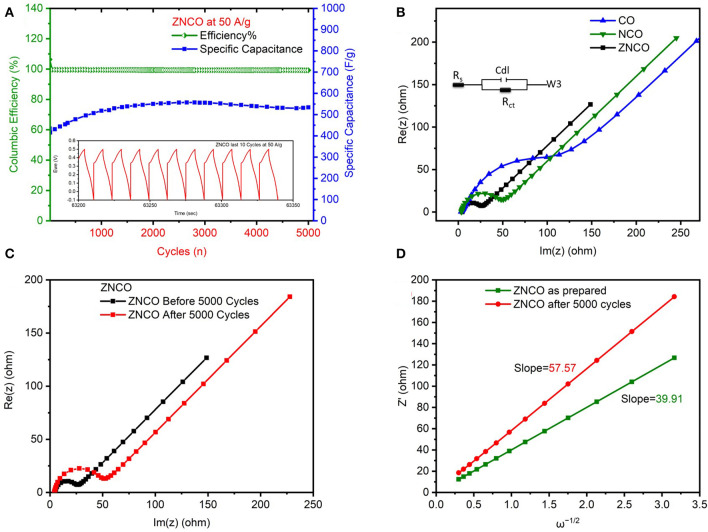
**(A)** Cyclic Stability and columbic efficiency of ZNCO nanostructure at 50 A g^−1^ **(B)** Nyquist plot of ZNCO, CO, NCO **(C)** Nyquist plot of ZNCO before and after cyclic performance **(D)** linear fits of the Z′ vs. ω^−1/2^.

The cyclic stability of developed electrode material was analyzed GCD cycles and the results are presented in Figure 5A and Figure S8. The ZNCO NWs exhibited excellent cyclic stability for over 5,000 charge/discharge cycles retaining over 120 % of initial capacitance at current density of 50 Ag^−1^. It is important to note that the capacitance increased for the first cycles because of activation of electrode materials whereby the extremely high current density limited the quick activation. Moreover, the Columbic Efficiency was almost ~100% after 5,000 cycles which shows excellent correlation between charge/discharge which is clearly evident from inset of Figure 5A. The EIS analysis was performed to evaluate the charge transfer resistance (R_ct_) as well as the series resistance (Rs). The ZNCO NWs exhibited lowest Rct value of 17.61 Ω as compared to ZCO, NCO, CO as shown in Figure 5B and Table S1. Relatively lower Rct values for ZNCO NWs clearly indicated that the ZNCO NWs is more conductive in comparison to other counterparts. The Rct values increased after cycling probably due to slighly change in morphology of material during the cyclic performance at high current density of 50 A g^−1^ as shown in Figure 5C. Furthermore, the diffusion coefficient of K^+^ ions (D_k_) was calculated by using low frequency region of Nyquist plot (Mahmood et al., 2019).

$$\begin{array}{l} D_{k}=0.5\frac{R^{2}T^{2}}{A^{2}n^{4}F^{4}C^{2}\sigma^{2}} \end{array}$$

Where T is the absolute temperature, R is the ideal gas constant, A is the surface area of electrode, n is number of electrons, F is faradays constant, C is the concentration of ions and σ is the Warburg coefficient. σ was calculated from linear plot of Z ' and ω^−1/2^ in Figure 5D. ZNCO NWs exhibits *D*_*k*_ of 1.04 × 10^−11^ which indicates enhanced diffusion of electrolyte ions through electrode materials. However, the electrolyte diffusion decreased slightly (4.99 × 10^−12^) after 5,000 cycles probably due to aggregation of particles upon cycling.

**Figure 6:**
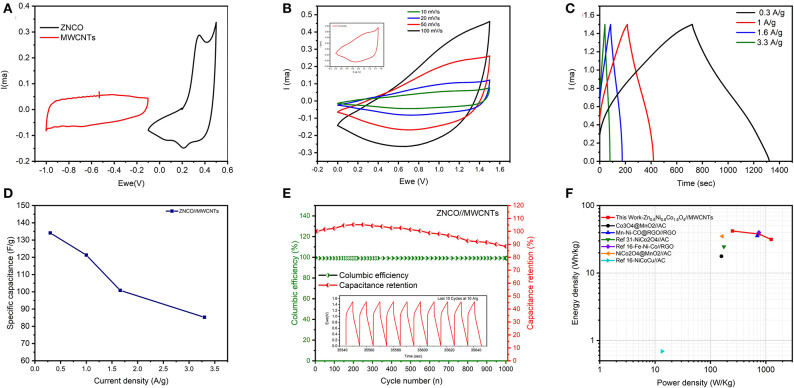
**(A)** CV curves of MWCNTs and ZNCO nanostructures at 10 mV s^−1^ **(B)** CV curves of ZNCO/MWCNTs ASC at different scan rates **(C)** Charge-discharge curves of ZNCO//MWCNTs asymmetric device at different current densities **(D)** specific capacitance of ZNCO//MWCNTs **(E)** Cyclic performance of ZNCO/MWCNTs at 10 A g^−1^ **(F)** Ragone plot of ZNCO//MWCNTs (this work), Co_3_O_4_@MnO_2_//AC (Huang et al., 2014), Mn-Ni-CO@RGO//RGO (Wu et al., 2017), NiCo_2_O_4_//AC (Zhong et al., 2012), Fe-Ni-Co//RGO (Sahoo et al., 2018), NiCo_2_O_4_@MnO_2_//AC (Xu et al., 2014), NiCoCu//AC (Sun et al., 2018).

To evaluate the performance of ZNCO NWs in SC device, the aqueous ASC was assembled using ZNCO NWs as positive electrode due to its high capacitance and MWCNTs as negative electrode due to their high power density. To obtain wide voltage window of 1.5 V, the operating voltage window of MWCNTs was adjusted to −1.0−0.1 V while the operating voltage window of ZNCO NWs was tested in the voltage window of −0.1–0.5 V as shown in Figure 6A. Moreover, prior to ASC testing, the capacitive performance of MWCNTs was investigated and presented in Figure S6. The CV and GCD analysis was used to investigate the capacitive performance of the assembled ASC (ZNCO NWs//MWCNTs) device and presented in Figures 6B,C. The CV analysis at different scan rates did not show any significant distortions clearly indicating good rate capability of the assembled device (Figure 6B). Moreover, the GCD show symmetric charge/discharge profile indicating ideal capacitive behavior. The ASC exhibited high specific capacitance of 121.26 F g^−1^ at 1 A g^−1^ and retained up to 85.2 F g^−1^ at 3.3 A g^−1^ in Figure 6D and Table S4. In addition, the assembled device exhibited high stability up to 1,000 cycles at current density of 10 A g^−1^ as shown in Figure 6E. An increase in capacitance was observed during first few cycles due to gradually improved wettability of the electrode materials which induce the participation of more active sites for charge storage. The ZNCO NWs//MWCNTs exhibited an overall 90 % capacitance retention after 1,000 charge/discharge cycles which clearly shows excellent stability of ZNCO NWs when applied in device as well. A slight loss of capacity can be ascribed to partial agglomeration of particles upon reversible cycling.

The energy and power density are important parameters in determining the application of ASC device. The ASC devices generally exhibit much lower energy densities (~0.1–10 Wh kg^−1^) which must be improved to realize high performance ASCs. The ZNCO NWs//MWCNTs ASC device exhibited high energy density of 37.89 Wh kg^−1^ at a corresponding power density of 749.8 W kg^−1^ which is higher/comparable to the reported literature as shown in Figure 6F. At a high-power density of 1249.94 W kg^−1^, the ASC device could retain up to 31.5 Wh kg^−1^ as seen in Ragone plot in Figure 6F. The higher energy density with corresponding higher power densities can be attributed to hierarchically porous structure of ZNCO NWs which allows large electrode/electrolyte interface and fast mass transport enabling operation at higher current densities and presence of more than one metallic sites whereby Zn and Ni not only stabilize the structure but also impart higher conductivity and decrease the overall cost of the product.

## Conclusions

In summary, the hierarchical mesoporous ZNCO NWs were successfully synthesized using two-step methodology (i.e., wet-chemical synthesis followed by high temperature calcination). As prepared ZNCO NWs were composed of small nanoparticles whereby hierarchical aggregation generated mesoporosity in the product. Owing to this mesoporous nature, the ZNCO NWs exhibited high surface area of 68.3066 m^2^ g^−1^. The ZNCO NWs exhibited outstanding electrochemical performance with a specific capacitance of 2082.21 F g^−1^ at 1 A g^−1^ with remarkable cyclic life of 5,000 cycles with capacitance retention of 120% owing to ternary metal oxide and hierarchically mesoporous structure. The asymmetric ASC device of ZNCO NWs//MWCNTs exhibited high specific capacitance of 121.26 F g^−1^ at 1 A g^−1^ as well as high energy density of 37.89 Wh kg^−1^. Superior electrochemical performance of ZNCO NWs//MWCNTs illustrates that the developed material provide opportunity to tailor new materials to meet the modern-day energy applications demand and the presented example could be one of the desired materials for next generation electrochemical devices.

## Data Availability Statement

All datasets generated for this study are included in the article/Supplementary Material.

## Author Contributions

The manuscript was written through contributions of all authors. All authors have given approval to the final version of the manuscript.

## Conflict of Interest

The authors declare that the research was conducted in the absence of any commercial or financial relationships that could be construed as a potential conflict of interest.
