# Supplementary material for: 3D Hierarchically Mesoporous Zinc-Nickel-Cobalt Ternary Oxide (Zn0.6Ni0.8Co1.6O4) Nanowires for High-Performance Asymmetric Supercapacitors
Source: Front Chem. 2020 Jun 15;8:487. doi: 10.3389/fchem.2020.00487 (PMC7307270; doi:10.3389/fchem.2020.00487)
Supplement: Supplementary file 1 [file Data_Sheet_1.pdf]

Supporting Information

3D Hierarchically Mesoporous Zinc-Nickel-Cobalt Ternary Oxide ($Zn_{0.6}Ni_{0.8}Co_{1.6}O_4$) Nanowires for High-Performance Asymmetric Supercapacitors

Muhammad Tayyab Ahsan,[a b] *Muhammad Usman,*[a] *Zeeshan Ali,*[a] *Rashad Ali,*[b] *Sofia Javed,*[a] *M. U. Farooq,*[c] *M. Aftab Akram,*[a]* *and Asif Mahmood,*[d]*

[a]School of Chemical & Materials Engineering, National University of Sciences and Technology (NUST), Islamabad, 44000, Pakistan

[b]Department of Materials Science and Engineering, College of Engineering, Peking University, 100871, Beijing, China

[c]School of Materials and Energy, University of Electronic Science and Technology of China, Chengdu, 611731, China

[d]Department of Physics, University of Education, Faisalabad Campus, 38000, Faisalabad, Pakistan

[e]School of Chemical and Biomolecular Engineering, The University of Sydney, 2006, Sydney, Australia

Corresponding authors:

Asif Mahmood (asif.mahmood@sydney.edu.au)

M. Aftab Akram (aftabakram@scme.nust.edu.pk)

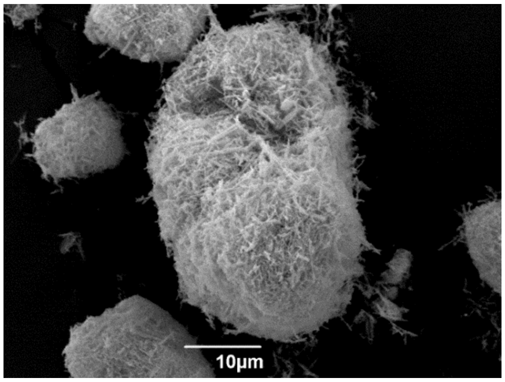

Figure S1. SEM of Cobalt oxide nanostructure (CO)

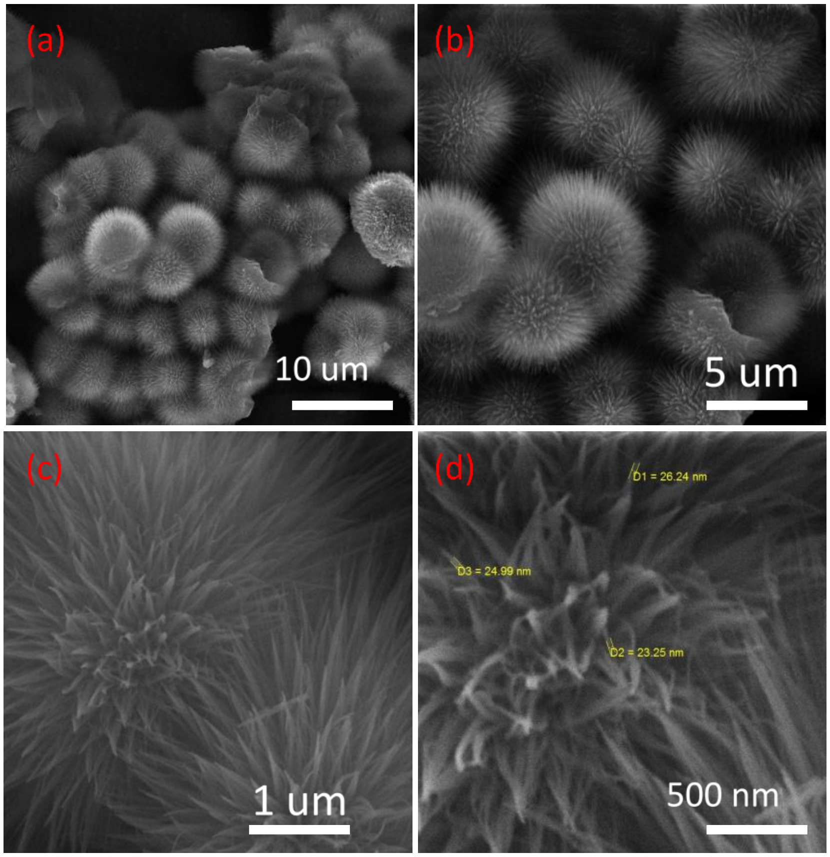

Figure S2. FESEM images of NCO nanostructures at low and high magnification

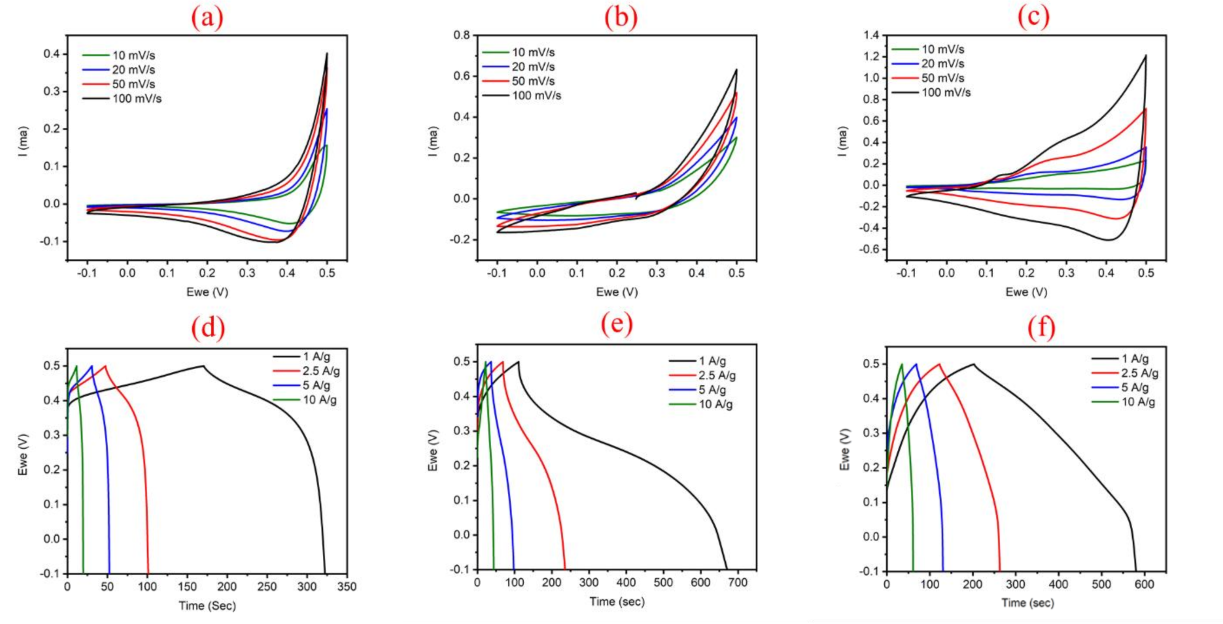

Figure S3. (a) CV curves of cobalt oxide (CO) at different scan rates (b) CV curves of nickel cobalt oxide (NCO) (c) CV curves of zinc cobalt oxide (ZCO) (d) GCD curves of cobalt oxide (CO) at different current densities (e) GCD curves of nickel cobalt oxide oxide (f) GCD curves of zinc cobalt oxide (ZCO)

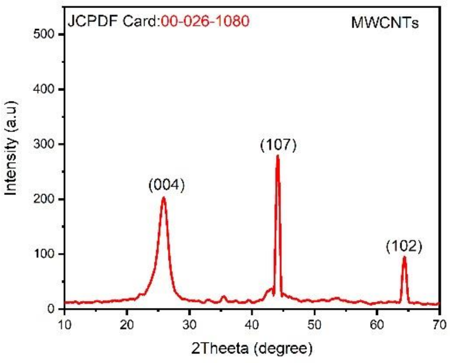

Figure S4. XRD of MWCNTs

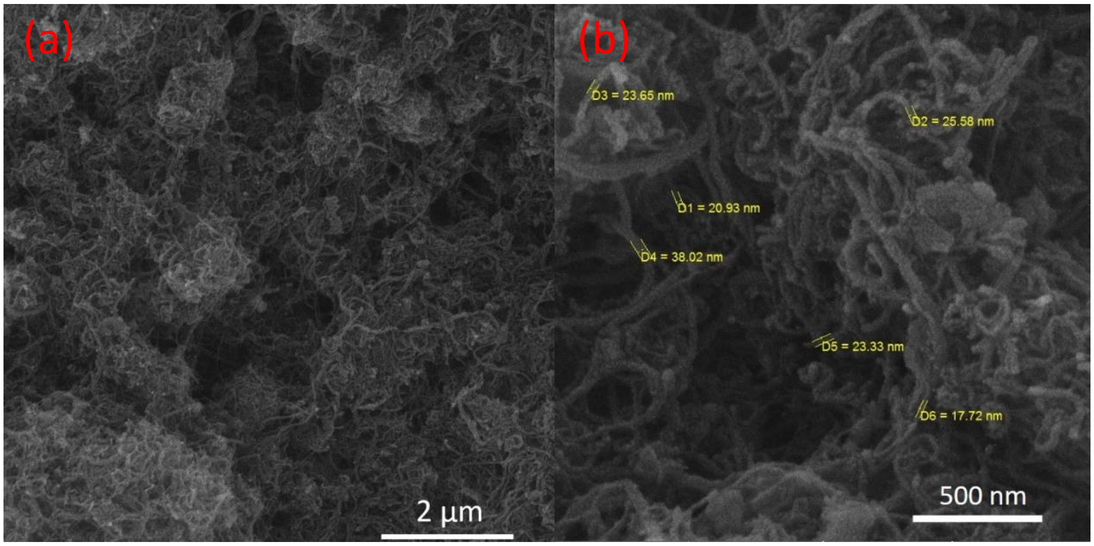

Figure S5. FESEM images of MWCNTs

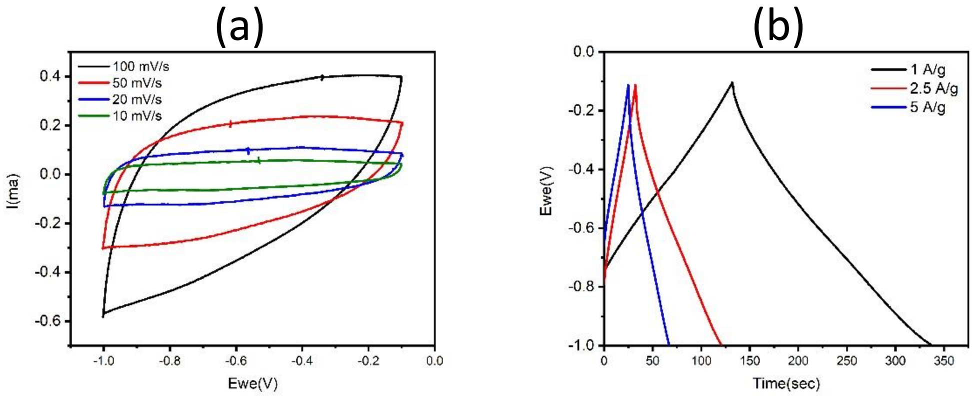

Figure S6. (a) CV curves of MWCNTs at different scan rate (b) GCD curves of MWCNTs at a different current density

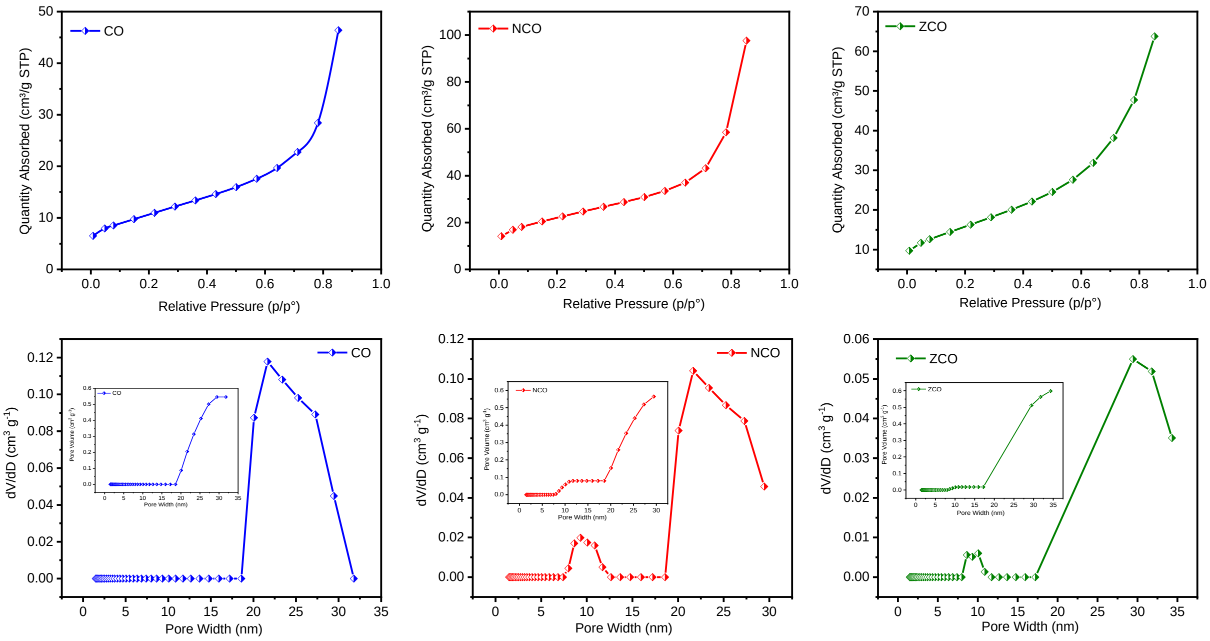

Figure S7. N_2 absorption isotherm of at 77k (a)Cobalt oxide (CO) (b) Nickel cobalt oxide (NCO) (c) Zinc cobalt oxide (ZCO)) BET pore volume and pore size distribution (d) CO (e) NCO and (f) ZCO

Cyclic performance and rate capability of Pure Cobalt oxide (CO) without doping is tested at and its gives 98% of capacitance retention after 1000 charge discharge cycles in Fig S8 whereas the ZNCO NWs shows better rate capability of 120% of retention after 5000 charge discharge cycles at 50 A g^{-1} .

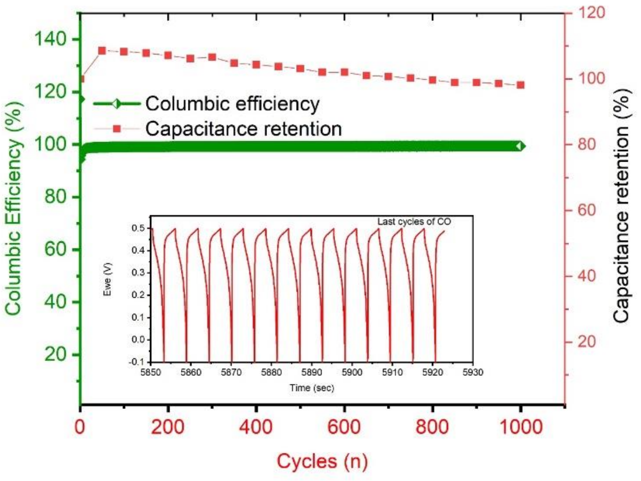

Figure S8. Cyclic performance and rate capability of CO

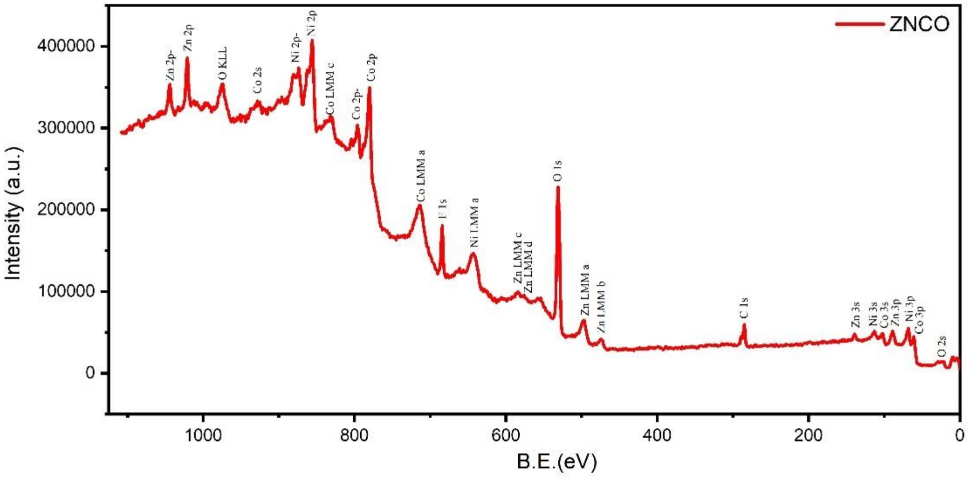

Figure S9. Low resolution XPS scan of Zinc Nickel Cobalt Oxide (ZNCO)

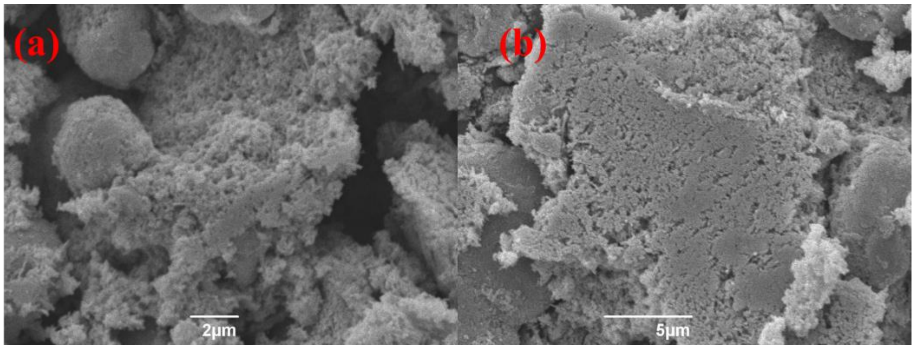

Figure S10. SEM images of ZNCO after GCD test

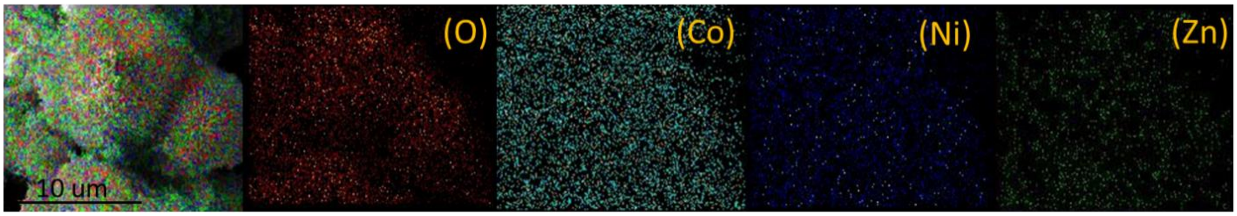

Figure S11. EDS mapping of ZNCO after GCD test

Table S1. Charge transfer Resistance of CO,NCO, ZCO & ZNCO

Sample	Charge transfer resistance Rct (Ohm)

ZNCO	17.61
NCO	36.74
CO	64.77
ZNCO after 5000 cycles	39.55

Table S2. Specific Capacitance of ZNCO,CO,NCO and ZCO at different current densities

**ZNCO**		**CO**		**NCO**		**ZCO**	
**Current Density**	Specific capacitance	Current Density	Specific capacitance	Current Density	Specific capacitance	Current Density	Specific capacitance
**A/g**	F/g	A/g	F/g	A/g	F/g	A/g	F/g
**1**	2082.21	1	253.55	1	936.18	1	630.43
**2.5**	1648.37	2.5	222.5	2.5	693.5	2.5	585.95
**5**	1383.86	5	180	5	509.83	5	516.66
**10**	1070	10	138.33	10	363.83	10	430.16

Table S3. Specific capacitance of ZNCO/MWCNTs at different current densities

Current density	Specific capacitance
A/g	F/g
0.3	134.14
1	121.268
1.6	100.83
3.3	85.2

Theoretical capacitance

Theoretical capacitance was calculated by using

$$C = Q/\Delta v$$

Where Q is the electrical energy per gram, C is the capacitance in Farad and Δv is the potential window at which electrochemical analysis were carried out

From EDS analysis of ZNCO $Zn_{0.6}Ni_{0.8}Co_{1.6}O_4$ and its molar mass is 244.028 g/mol and power to active 1 mol of material is 1 Farad which is equal to 96485 C.

C= $\frac{96485 \times (0.6\times 65.38 + 0.8\times 58 + 1.6 \times 58.93 + 64)}{0.6}$

C=$\frac{96485\times(244.028)}{0.6}$

C=1976.92F/g

Theoretical capacitance is comparable with practical calculated from GCD curves. This is slightly lower than practical value which is 2082.2 F/g.This may be due to the reason of redox reactions at the interface.

Table S4. Comparsion of different studies of Cobalt oxides nanostructured

Material	Morphology	Asymmetric device	Specific Capaitance	Voltage Window Volts	Current Density	Energy Density Wh/kg	Reference
**$NiCo_2O_4/MnO_2$**	Nanowires	Activated carbon	112 F/g	0-1.5	1 A/g	35	[1]
**$NiCo_2O_4@MnO_2$**	Nanowires	Activated Carbon	161F/g	0-1.3	2.5 mA/cm^2	37.8	[2]
**$NiCo_2O_4@NiO$**	Nanowires	Activated carbon	----	0-1.4	1 A/g	31.5	[3]
**RGO@Mn-Ni-Co**	Nanosheets	RGO	------	0-1.4	1 A/g	35.6	[4]
**$FeCo_2O_4@GF$**	Heirarchical nanostructure	PANI@graphene	61.58 mF/cm^2	0-1.4	0.1 mA/cm^2	16.76 $\mu Wh/cm^2$	[5]
**Zn-Ni-Co**	Nanowires	Activated carbon	113.9 F/g	0-1.5	1 A/g	35.6	[6]
**This work $Zn_{0.6}Ni_{0.8}Co_{1.6}O_4$**	Hierarchical Nanowires	MWCNTs	121.268 F/g	0-1.5	1 A/g	37.89	

References

[1] K. Xu *et al.*, "Hierarchical mesoporous NiCo2O4@MnO2 core–shell nanowire arrays on nickel foam for aqueous asymmetric supercapacitors," *Journal of Materials Chemistry A*, vol. 2, no. 13, p. 4795, 2014.

[2] W. Ma, H. Nan, Z. Gu, B. Geng, and X. Zhang, "Superior performance asymmetric supercapacitors based on $ZnCo_2O_4$@MnO_2 core–shell electrode," *Journal of Materials Chemistry A*, vol. 3, no. 10, pp. 5442–5448, 2015.

[3] X. Liu, J. Liu, and X. Sun, "$NiCo_2O_4$@NiO hybrid arrays with improved electrochemical performance for pseudocapacitors," *Journal of Materials Chemistry A*, vol. 3, no. 26, pp. 13900–13905, 2015.

[4] C. Wu, J. Cai, Y. Zhu, and K. Zhang, "Hybrid Reduced Graphene Oxide Nanosheet Supported Mn–Ni–Co Ternary Oxides for Aqueous Asymmetric Supercapacitors," *ACS Applied Materials & Interfaces*, vol. 9, no. 22, pp. 19114–19123, Jun. 2017.

[5] J. Zhao *et al.*, "Hierarchical ferric-cobalt-nickel ternary oxide nanowire arrays supported on graphene fibers as high-performance electrodes for flexible asymmetric supercapacitors," *Nano Research*, vol. 11, no. 4, pp. 1775–1786, Apr. 2018.

[6] C. Wu *et al.*, "Hierarchical Mesoporous Zinc–Nickel–Cobalt Ternary Oxide Nanowire Arrays on Nickel Foam as High-Performance Electrodes for Supercapacitors," *ACS Applied Materials & Interfaces*, vol. 7, no. 48, pp. 26512–26521, Dec. 2015.
